# EphA2-receptor deficiency exacerbates myocardial infarction and reduces survival in hyperglycemic mice

**DOI:** 10.1186/s12933-014-0114-y

**Published:** 2014-08-13

**Authors:** Augustin DuSablon, Susan Kent, Anita Coburn, Jitka Virag

**Affiliations:** Department of Physiology, Brody School of Medicine, East Carolina University, 600 Moye Blvd, Greenville, NC 6N-98 USA; Department of Comparative Medicine, Brody School of Medicine, East Carolina University, 600 Moye Blvd, Ed Warren Life Sciences Building, Greenville, NC USA

**Keywords:** Diabetes, Ischemia, Myocardial infarction, Hyperglycemia, EphrinA1/EphA

## Abstract

**Background:**

We have previously shown that EphrinA1/EphA expression profile changes in response to myocardial infarction (MI), exogenous EphrinA1-Fc administration following MI positively influences wound healing, and that deletion of the EphA2 Receptor (EphA2-R) exacerbates injury and remodeling. To determine whether or not ephrinA1-Fc would be of therapeutic value in the hyperglycemic infarcted heart, it is critical to evaluate how ephrinA1/EphA signaling changes in the hyperglycemic myocardium in response to MI.

**Methods:**

Streptozotocin (STZ)-induced hyperglycemia in wild type (WT) and EphA2-receptor mutant (EphA2-R-M) mice was initiated by an intraperitoneal injection of STZ (150 mg/kg) 10 days before surgery. MI was induced by permanent ligation of the left anterior descending coronary artery and analyses were performed at 4 days post-MI. ANOVAs with Student-Newman Keuls multiple comparison post-hoc analysis illustrated which groups were significantly different, with significance of at least p < 0.05.

**Results:**

Both WT and EphA2-R-M mice responded adversely to STZ, but only hyperglycemic EphA2-R-M mice had lower ejection fraction (EF) and fractional shortening (FS). At 4 days post-MI, we observed greater post-MI mortality in EphA2-R-M mice compared with WT and this was greater still in the EphA2-R-M hyperglycemic mice. Although infarct size was greater in hyperglycemic WT mice vs normoglycemic mice, there was no difference between hyperglycemic EphA2-R-M mice and normoglycemic EphA2-R-M mice. The hypertrophic response that normally occurs in viable myocardium remote to the infarct was noticeably absent in epicardial cardiomyocytes and cardiac dysfunction worsened in hyperglycemic EphA2-R-M hearts post-MI. The characteristic interstitial fibrotic response in the compensating myocardium remote to the infarct also did not occur in hyperglycemic EphA2-R-M mouse hearts to the same extent as that observed in the hyperglycemic WT mouse hearts. Differences in neutrophil and pan-leukocyte infiltration and serum cytokines implicate EphA2-R in modulation of injury and the differences in ephrinA1 and EphA6-R expression in governing this are discussed.

**Conclusions:**

We conclude that EphA2-mutant mice are more prone to hyperglycemia-induced increased injury, decreased survival, and worsened LV remodeling due to impaired wound healing.

## Background

Diabetes affects nearly 6% of the US population and about 30% of acute coronary syndrome patients hospitalized have diabetes [1]. In 2004, 68% of diabetes-related deaths were associated with heart disease. Morbidity, mortality, and re-infarction rates are 2-4 times higher following MI in diabetic than in non-diabetic subjects, with one-year mortality in this population as high as 50% [2]. Despite the evidence for improved outcomes from cardiovascular disease in the general population over the past three decades, these benefits have not been paralleled in the diabetic population since the diabetic heart is refractory to known cardioprotective approaches. The heart, being a terminally differentiated organ, lacks endogenous regenerative capacity and thus, developing techniques to maximize myocardial salvage post MI continues to be a health care priority.

The Eph receptors and their cognate ligands, the ephrins are the largest family of receptor tyrosine kinases (RTKs) and their signaling has been implicated in various cellular processes during embryogenesis, tumorigenesis, vasculogenesis and in the regulation of inflammation and apoptosis [3–12]. Previous research has shown that exogenous administration of EphrinA1-Fc following an ischemic event reduces cardiac injury by reducing cardiomyocyte death and inflammation, and promoting tissue regeneration [13–15]. To investigate whether or not ephrinA1-Fc also has potential therapeutic value in the hyperglycemic heart, it is first necessary to compare and contrast ephrinA/EphA expression in WT hyperglycemic and normoglycemic myocardium versus hyperglycemic and normoglycemic EphA-2-R-M myocardium following an MI. We hypothesize that EphrinA1/EphA signaling is differentially and unfavorably influenced by hyperglycemia in EphA2-R-M mice and that injury, remodeling, and dysfunction are further exacerbated by hyperglycemia.

Our results indicate that in mice lacking a functional EphA2-R, hyperglycemia worsens remodeling and cardiac function post-MI and this is due to an aggravated inflammatory response, poor infarct resolution, and inadequate healing. This is at least in part due to insufficient production of endogenous protective ephrinA1 in both normoglycemic and hyperglycemic WT and EphA2-R-M mice as well as increased EphA6 expression in EphA2-R-M compared to WT mice. We conclude that exogenous administration of EphrinA1 would be of therapeutic value in the hyperglycemic heart and EphA2 is necessary for appropriate signal transduction. Further studies to assess this are in progress.

## Methods

### Ethical approval

All procedures were approved by the East Carolina University Institutional Animal Care and Use Committee and the investigation conforms to the Guide for the Care and Use of Laboratory Animals published by the US National Institutes of Health.

### Animals

WT129SF2/J mice (stock #101045) (WT) and WT129S6-Epha2^tm1Jrui^/J (stock #006028; a pan-knockout; EphA2-R-M) mice aged 8-12 weeks were used in these experiments. Male mice were housed in individually ventilated cages and animal care was maintained by the Department of Comparative Medicine at The Brody School of Medicine at East Carolina University. Mice were exposed to 12 h/12 h light/dark cycle conditions and received food and water ad libitum.

### Blood glucose

WT and EphA2-R-M mice were given an intraperitoneal injection of 150 mg/kg of streptozotocin (STZ) or 0.1 mol/l of citrate buffer. Mice became diabetic (as evidence by polyuria and polydipsia) at least 1 week prior to surgery. Ten days after the STZ/citrate injection, but prior to surgery, mice were fasted for 4 hours and blood glucose levels were measured (OneTouch Ultra glucometer, Lifescan, CA, USA). Mice were considered hyperglycemic if blood glucose was greater than 200 mg/dl. There were four groups of control mice and 4 groups of infarcted mice (WT citrate, WT STZ, EphA2-R-M citrate, and EphA2-R-M STZ) from which tissue was collected for analysis 4 days post-MI.

### Surgical procedure, cardiac function and tissue collection

Myocardial infarction was induced by permanently ligating the left coronary artery in anesthetized, mechanically ventilated mice. Briefly, male WT and EphA2-R-M mice (8-12 weeks) were anesthetized with an intraperitoneal (IP) injection of 20 μl/g body weight Avertin (20 mg/ml), a left thoracotomy performed, and the left anterior descending coronary artery was permanently occluded using an 8-0 suture. The rib cage, muscle, and skin were then closed with a 6-0 suture. Uninjured sham controls underwent the entire procedure including suture pass under the coronary [13,16].

Echocardiography was performed on conscious mice at 4 days post-MI. A VisualSonics Vevo 2100 diagnostic ultrasound, using M-mode and 30 MHz probe, was used to obtain LV dimensions in diastole and systole. End-diastolic measurements of interventricular septal thickness (IVSd), left ventricular posterior wall thickness (LVPWd), and left ventricular internal diameter (LVIDd) were obtained at the point of maximal LV diastolic dimension. End-systolic dimensions (IVSs, LVPWs, and LVIDs) were measured at the time of most anterior systolic excursion of the LVPW associated with minimal chamber dimension. Average measurements were calculated using the leading-edge technique of 3- to 5-consecutive sinus beats. Ejection fraction (EF) was calculated from LV dimensions above using the following formula: (LVIDd^3^ – LVIDs^3^)/LVIDd^3^ × 100%. FS was calculated using the formula (LVIDs-LVIDs)/LVIDd × 100% [17].

At the time of sacrifice, mice were anesthetized with an IP injection of 0.1 mL pentobarbital (390 mg/mL) and a pneumothorax was performed. Hearts to be used for histology and immunohistochemistry were arrested in diastole using cold KCl (30 mM), excised, rinsed in PBS, and immersed in zinc fixative. For RNA and protein analyses, the right ventricle was dissected away from the heart and discarded while the remaining left ventricle was snap frozen in liquid nitrogen.

### Histology, morphometry, and immunohistochemistry

Tissue was harvested and immersed in zinc fixative, then processed and embedded in paraffin as per routine procedures. For immunohistochemical staining and morphometric measurements, whole hearts were transversely sectioned into 4 slices of equal thickness, processed in an automated tissue processor (TP1020, Leica, Nußloch, Germany), and embedded in paraffin (MICROM EC350, Richard-Allan Scientific, Kalamazoo, MI, USA) [18,19]. Blocks were then sectioned into 5um sections and mounted on Superfrost Plus glass slides for histologic or immunohistochemical staining.

Morphometric measurements of the left ventricle, infarct area, and chamber area were performed on tissue sections 4 days post-MI (n = 7-12/group). Images of 4 hematoxylin and eosin (H&E)-stained sections of each heart were taken at × 20 magnification using a DP70 digital camera. Myocyte cross-sectional area (MCSA) was measured in 3-8 cardiomyocytes with centrally located nuclei in each of 6 images (600×) in the endocardium and epicardium (n = 3-5/group) using Scion imaging software (Scion Corporation, Frederick, MD, USA).

Slides stained with picrosirius red for fibrillar collagen and fast green for contrast were used to quantify interstial fibrosis [20]. Adobe Photoshop software was used to count the red collagen fibril pixels which were expressed as a percentage of the total number of pixels (total = green + red - white) in five images at 400× taken in each of 2 cross sections per heart remote to the infarct but in the same plane.

Tissue sections were deparaffinized in xylene and endogenous peroxidases quenched with 3% H_2_O_2_ in methanol. As described elsewhere previously, slides were rinsed in PBS and incubated with Ly6G (BD Biosciences, #550291) to assess neutrophil infiltration, CD45 (BD Biosciences; #550539) for macrophage density [13,19–22]. Isolectin B4 (Vector Labs;B-1205) or CD31 (BD Biosciences, #553371) was used to measure capillary density in uninjured control and infarcted hearts respectively [19]. Slides were incubated with appropriate biotinylated secondary antibodies and then with Avidin Biotin Complex (Vector Labs PK-6100). The reaction product was visualized with DAB (Vector, SK-4100), counterstained with methyl green, dehydrated in xylene, and slides were coverslipped.

### ELISA

A mouse cytokine proteome profiler array (R&D #ARY006) was used to compare levels of 40 serum cytokines as per manufacturer’s protocol. Forty microliters from each of 5 different samples were combined for each group. The samples were run in duplicate and data are expressed as relative expression (pixel density = relative units) with levels appropriately normalized to internal reference spot controls.

### qRT-PCR

Whole left ventricles from mice 4 days post-MI were homogenized using Trizol for RNA isolation. Purification was performed using the Qiagen RNeasy kit. cDNA was made for each sample using a high capacity cDNA kit. Real-time PCR (qRT-PCR) performed using an Applied Biosystems thermocycler. TaqMan primers were obtained from Applied Biosciences (ephrinA1: Mm00438660_m1, EphA1: Mm00445804_m1, EphA3: Mm00580743_m1, EphA4: Mm00433056_m1, EphA5: Mm00433074_m1, EphA6: Mm00433094_m1, EphA7: Mm00833876_m1, GAPDH: Mm99999915_g1). All samples were run in triplicate and a reaction mixture of 10 μl (100 ng RNA) was amplified using recommended conditions from Applied Biosciences. Gene expression was normalized to expression of GAPDH. Fluorescence data were analyzed using the ∆∆ Ct method.

### Western blotting

Whole left ventricles of uninjured sham control hearts and hearts 4 days post-MI from each of the 8 groups of mice were homogenized in a lysis buffer containing 50 mM Hepes, 10 mM EDTA, 100 mM NaF, 50 mM sodium pyrophosphate, 1% protease, and 1% phosphatase inhibitors. The Bradford Assay was used to quantify the amount of protein. Western blotting was performed on a 4–12% gradient Bis-Tris gel (BioRad) in 1× Mops running buffer.

Fifty micrograms of sample (n = 3/group) was loaded per well. The gel was run for 1 h at 155 V, and transferred onto pure nitrocellulose membranes (BioRad). Antibodies: GAPDH (Millipore, #MAB374), NF-*κ*B (Santa Cruz, #sc-372), EphA6-R (SantaCruz #25740), MMP-9 (R&D, BAF909), p-mTOR (Cell Signaling, #5536), mTOR (Cell Signaling, #2983), and ephrinA1 (Santa Cruz, #sc-911) were followed by appropriate secondary antibodies. All blots were detected with Immun-Star Horseradish peroxidase chemiluminescence (Bio-Rad, Hercules, CA) and imaged on an Alpha Innotech imaging system (ProteinSimple, San Jose, CA). Densitometry was performed using Image J 1.42 software (NIH, Bethesda, MD) and the intensity of each protein was normalized to GAPDH.

### Statistics

ANOVA (analysis of variance) with Student-Newman Keuls multiple comparison post-hoc analysis illustrated which groups were statistically different, with significance of at least p < 0.05.

## Results

### Survival

**Figure 1:**
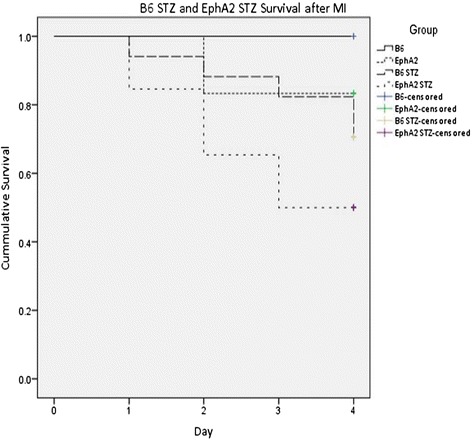
**Cumulative Survival in WT STZ and EphA2-R-M STZ mice 4 days post-MI.** Survival of EphA2-R-M mice post-MI with or without STZ is less than that of their WT counterparts (p < 0.05).

The Kaplan-Meier graph in Figure 1 depicts the survival rates during the first 4 days post-MI. Mice subjected to STZ + MI had lower cummulative survivals than animals with only MI. WT (n = 5) and EphA2-R-M (n = 6) control animals had a cummulative surival of 100% and 83.3% 4 days post-MI, respectively. WT STZ (n = 17) had a cummulative survival of 70.6% 4 days post-MI and EphA2-R-M STZ (n = 26) had a cummulative survival of 50% after MI.

### Infarct size

**Figure 2:**
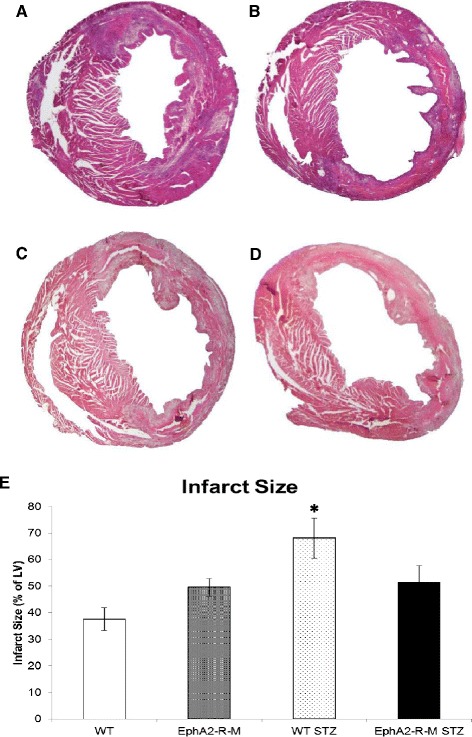
**Infarct size in WT and EphA2-R-M Normo-and Hyperglycemic mice.** Representative images of H&E stains (x20) of normoglycemic WT mouse heart **(A)** and EphA2-R-M **(B)**, and hyperglycemic WT **(C)** and EphA2-R-M **(D)** mouse hearts. Infarct size **(E)** in WT mice was 45% larger in WT hypergylcemic mice compared with normoglycemic mice (p < 0.01). EphA2-R-M mice had slightly with slightly larger infarcts but this was not exacerbated by hyperglycemia **(E)**.

**Figure 3:**
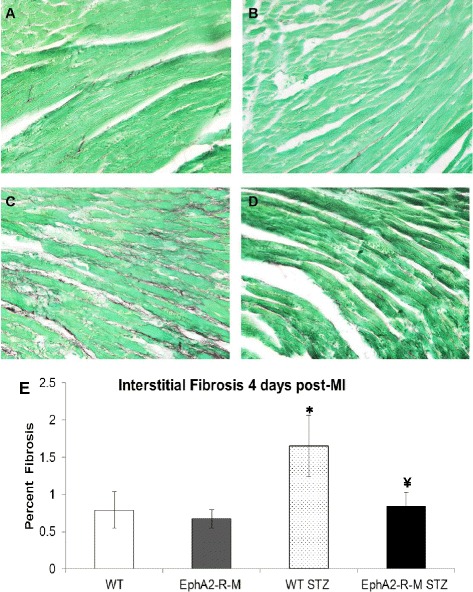
**Interstitial Fibrosis.** Representative images (x400) of normoglycemic WT mouse heart **(A)** and EphA2-R-M **(B)**, and hyperglycemic WT **(C)** and EphA2-R-M **(D)** mouse hearts stained with picrosirius red and fast green. Percent interstitial fibrosis **(E)** was elevated in hyperglycemic WT mice 4 days post-MI (*, p < 0.05) compared to normoglycemic WT hearts and hyperglycemic EphA2-R-M mouse hearts (¥, p < 0.01).

Infarct size 4 days post-MI is shown in Figure 2. 4 days post-MI, the infarct size in WT mouse hearts (n = 12) was 37.5 ± 4.3% of the left ventricle compared with 49.5 ± 3.3% in EphA2-R-M mice (n = 10). In hyperglycemic WT mice, infarct size at 4 days post-MI was 68% ± 7.7% (n = 8) compared with 51.5 ± 6.2% in EphA2-R-M (n = 9) mice. There were no differences between LV and chamber area at 4 days post-MI compared to hyperglycemic mice of both WT and EphA2-R-M mice at 4 days post-MI. There were no differences in endocardial myocyte cross-sectional area (MCSA) among the 8 groups studied. STZ-treated groups showed a nonsignificant trend to have decreased epicardial MCSA. Epicardial cardiomyocytes in EphA2-R-M mice demonstrated a non-significant trend to hypertrophy post-MI (263.9 ± 15.4 vs 315.0 ± 30.4) but this was blunted in the STZ-treated EphA2-R-M mice (203.8 ± 8.9 vs 222.3 ± 21.1). Similarly, there was an increase in the interstitial fibrosis in the remote myocardium in normoglycymic mice post-MI and this increased nearly 2-fold in hyperglycemic WT mice but did not change in hyperglycemic EphA2-R-M mice (representative images Figure 3A-D; numerical Figure 3E).

### Ly6-G^+^ neutrophil density

#### STZ

**Figure 4:**
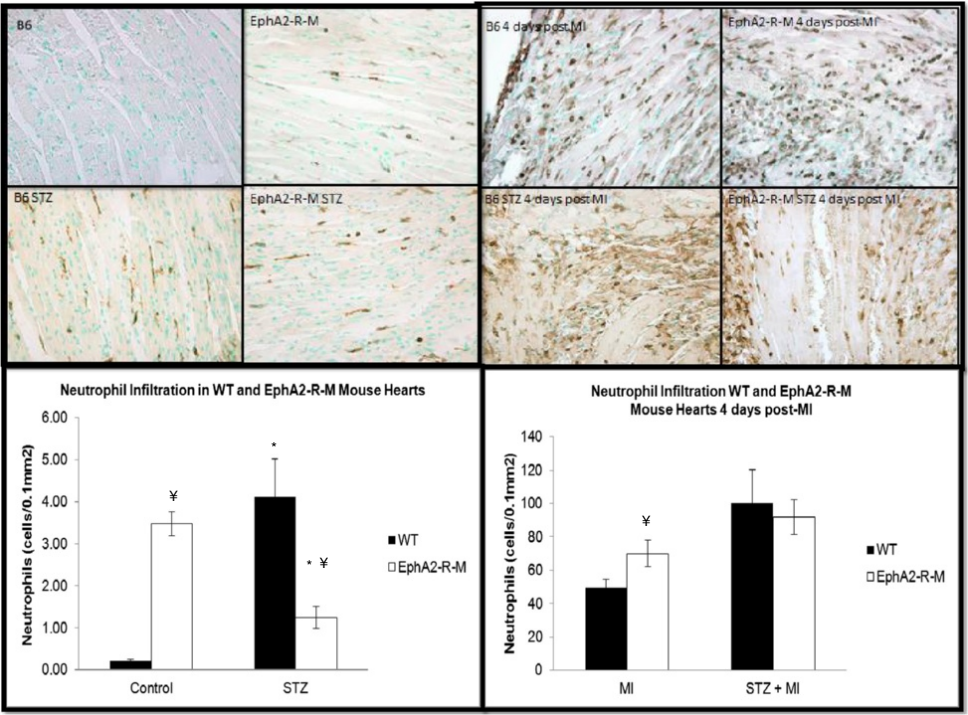
**Neutrophil Infiltration in WT STZ and EphA2-R-M STZ hearts.** Neutrophil density in the infarct zone before (top) and after (bottom) MI. *Different from control, p < 0.001; ¥ Different from WT, p < 0.001.

Ly6-G^+^ cell counts to determine neutrophil density in control and STZ-treated WT and EphA2-R-M mouse hearts is shown in Figure 4. Neutrophil density in normoglycemic WT (n = 6) hearts was 0.21 ± 0.04 cells/0.1 mm^2^ compared with the normoglycemic EphA2-R-M (n = 3) hearts with 3.48 ± 0.29 cells/0.1 mm^2^ (p < 0.01). In hyperglycemic mouse hearts we observed a neutrophil density of 4.10 ± 0.91 cells/0.1 mm^2^ in WT STZ (n = 6) hearts and 1.24 ± 0.25 cells/0.1 mm^2^ in EphA2-R-M STZ (n = 7) hearts (p < 0.05). Neutrophil density in WT STZ was nearly 19-fold higher than WT controls (p < 0.01). EphA2-R-M STZ hearts however, had 64% less neutrophils than EphA2-R-M controls (p < 0.01).

#### STZ + MI

Ly6-G^+^ staining for neutrophil density in the infarct zone 4 days post-MI (Figure 4) showed an increase in EphA2-R-M (70.04 ± 7.86 cells/0.1 mm^2^; n = 9) compared with WT (49.38 ± 5.26 cells/0.1 mm^2^; n = 10) mouse hearts (p < 0.05). Neutrophil density 4 days post-MI was not different between the WT + STZ mouse hearts (92.89 ± 23.34 cells/0.1 mm^2^) and the EphA2-R-M + STZ post-MI (91.89 ± 10.25 cells/0.1 mm^2^). In WT mice, MI alone increased neutrophil infiltration 103% over infarcted EphA2-R-M mice (p = 0.06). STZ + MI only increased neutrophils by a non-significant 31% in EphA2-R-M compared to STZ + MI WT hearts (p = 0.12).

### CD45^+^ macrophage density

#### STZ

**Figure 5:**
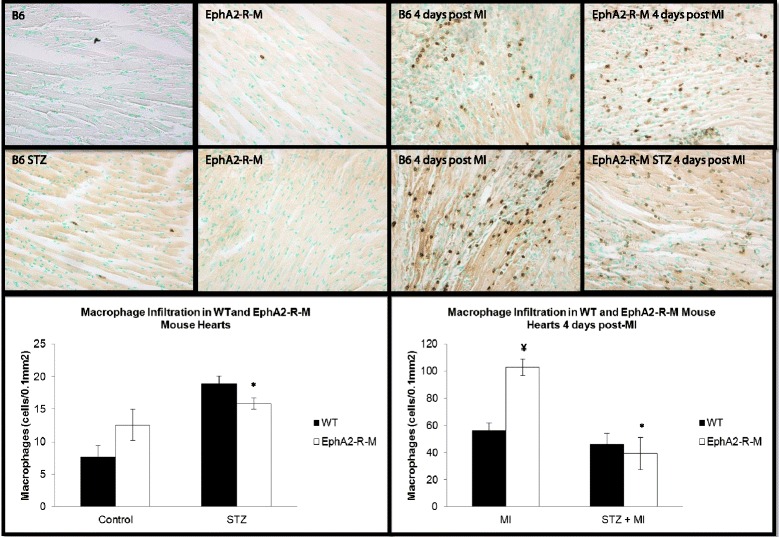
**Macrophage Infiltration in WT STZ and EphA2-R-M STZ hearts.** Macrophage density in the infarct zone of control hearts (top; *Different from control, p < 0.001; ¥ Different from WT, p < 0.001) and post-MI (bottom; *Different from MI, p < 0.001; ¥ Different from WT, p < 0.001).

Macrophage density in normoglycemic hearts was no different between WT (n = 3) and EphA2-R-M (n = 10) (Figure 5). CD45^+^ staining of macrophage infiltration in WT STZ (n = 6) hearts was 18.86 ± 1.22 cells/0.1 mm^2^ compared with the EphA2-R-M STZ (n = 6) with 15.85 ± 0.91 cells/0.1 mm^2^ (p = 0.07). There was a 147% increase in macrophage infiltration in WT STZ (n = 6) compared with WT controls (n = 3) (p < 0.01). In EphA2-R-M STZ (n = 6), there was an insignificant increase of only 26% in macrophage infiltration compared with EphA2-R-M controls (p = 0.22).

#### STZ + MI

Macrophage density 4 days post-MI (Figure 5) in normoglycemic WT (n = 7) hearts was 55.97 ± 5.94 cells/0.1 mm^2^ compared with the normoglycemic EphA2-R-M (n = 8) hearts with 102.76 ± 5.94 cells/0.1 mm^2^ (p < 0.001). Macrophage density was no different after MI in WT STZ (n = 5) and EphA2-R-M STZ (n = 5) hearts (p = 0.65). Macrophage infiltration was 17% less in WT STZ (n = 5) hearts after MI compared with WT (n = 7) hearts (p = 0.34). However, EphA2-R-M STZ hearts post-MI (n = 5) had 64% less macrophages than EphA2-R-M hearts after MI (n = 8) (p < 0.01).

### Serum cytokines

**Figure 6:**
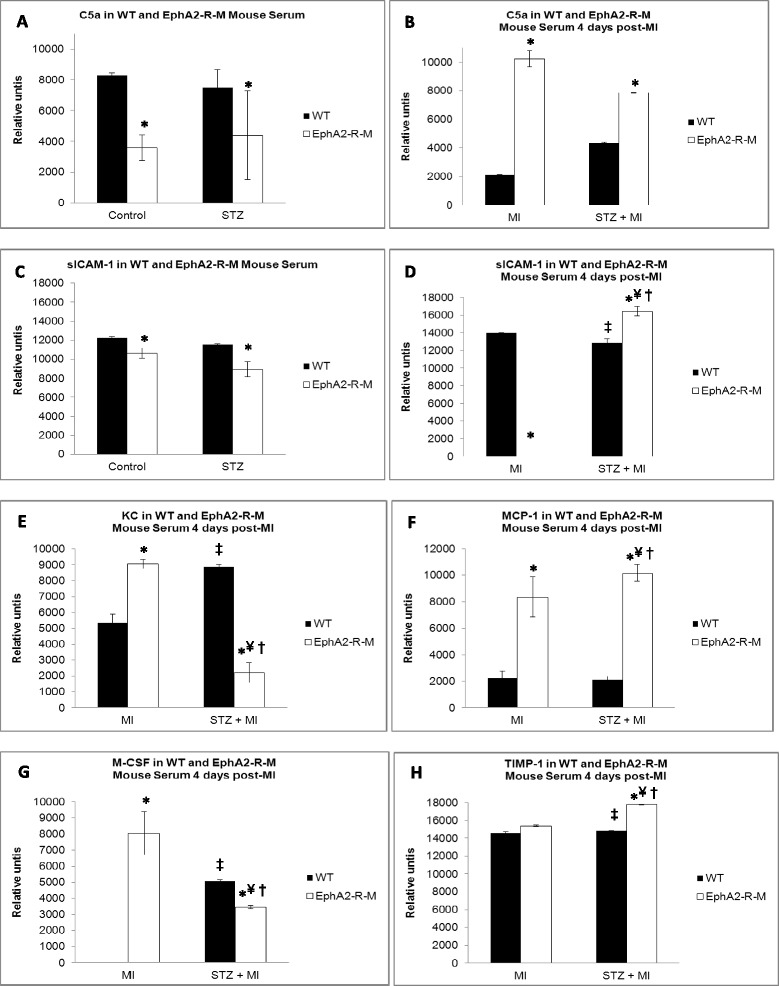
**Serum Cytokines in Normoglycemic and Hyperglycemic WT and EphA2-R-M mice. Panel A** and **C** show the differences in C5a and sICAM-1 between normoglycemic and hyperglycemic WT and EphA2-R-M mice. **Panel B** and **D** shows how C5a and sICAM-1 change post-MI. **Panels E-H** show post-MI changes in WT versus EphA2-R-M mice in serum levels of KC, MCP-1, M-CSF, and TIMP-1. (p < 0.05: *different from WT, ^●^different from WT STZ, ^‡^different from WT 4d, ^¥^different from EphA2-R-M 4d, ^†^different from WT 4d STZ).

Serum cytokine levels are shown in Figure 6. Of the 40 cytokines tested by the array, only 10 were expressed in the samples tested and 4 of these, TREM, TNF-α, IL-1α, and MIG were only detected in the EphA2-R-M 4d + STZ group (data not shown).

C5a and sICAM-1 were present in all groups (panels A - D) whereas M-CSF, MCP-1, KC, and TIMP-1 were only detected in injured hearts (panels E- H).

### Capillary density

#### STZ

**Figure 7:**
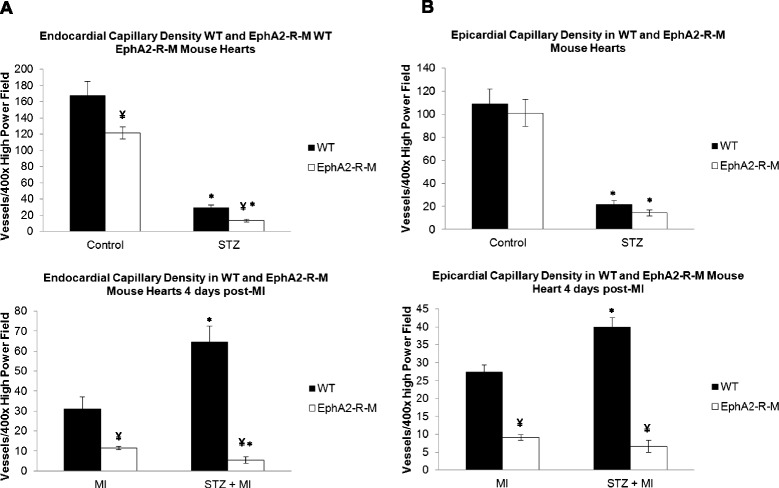
**Endocardial (A) and Epicardial (B) Capillary Density in WT and EphA2-R-M hearts. A**: The top graph shows the capillary density in the endocardium of uninjured control hearts from normoglycemic (left) and hyperglycemic (right; STZ) hearts (top; *Different from control, p < 0.001; ¥ Different from WT, p < 0.01) and hearts 4 days post-MI (bottom; *Different from MI, p < 0.001; ¥ Different from WT, p < 0.01). **B**: The top graph shows the capillary density in the epicardium of uninjured control hearts from normoglycemic (left) and hyperglycemic (right; STZ) hearts (*Different from control, p < 0.001) and hearts 4 days post-MI (bottom; *Different from MI, p < 0.01; ¥ Different from WT, p < 0.001).

Endocardial capillary density in WT control (n = 6) hearts was 167 ± 18 vessels per 400x high power field and 121 ± 8 vessels per 400x high power field in EphA2-R-M control (n = 5) hearts (p < 0.05). In WT STZ hearts (n = 5), endocardial capillary density was reduced by 82% compared with control hearts (p < 0.001). EphA2-R-M STZ hearts (n = 5) observed a decrease in capillary density in the endocardium by 89% compared with WT control hearts (p < 0.001). EphA2-R-M STZ (n = 5) hearts had 54% less capillaries in the endocardium than WT STZ (n = 5) hearts (p < 0.01). These results are shown in Figure 7A.

#### STZ + MI

Endocardial CD31^+^ staining 4 days post-MI showed that capillary density was reduced by 63% in normoglycmic EphA2-R-M (n = 6) hearts compared with WT (n = 4) (p < 0.05). After MI, WT STZ (n = 5) had an increase in capillary density of 109% compared with WT control (n = 4) hearts. (p < 0.05). EphA2-R-M STZ (n = 4) had 53% less capillaries than EphA2-R-M control (n = 6) hearts after MI (p < 0.05). EphA2-R-M STZ (n = 4) had 92% less capillary density than WT STZ (n = 5) after infarction (p < 0.01). These results are depicted in Figure 7A.

Epicardial capillary density in WT control (n = 6) hearts was 108.97 ± 12.98 vessels per 400x high power field and 100.90 ± 11.71 vessels per 400x high power field in EphA2 control (n = 5) hearts (p = 0.65). In WT STZ hearts (n = 5), epicardial capillary density was reduced by 80.3% compared with WT control hearts (p < 0.001). EphA2 STZ hearts (n = 5) observed a decrease in capillary density in the epicardium by 86% compared with EphA2-R-M control hearts (p < 0.001). EphA2 STZ (n = 5) hearts had 33.3% less capillaries in the epicardium than WT STZ (n = 5) hearts (p = 0.15). These results are shown in Figure 7B.

Epicardium CD31^+^ staining 4 days post-MI was 27.38 ± 1.99 vessels per 400x high power field in WT control (n = 4) hearts and 9.04 ± 0.73 vessels per 400× high power field in EphA2 (n = 6) control hearts after MI (p < 0.01). WT STZ (n = 5) had an increase in epicardial capillary density of 46% compared with WT (n = 4) hearts after infarction (p < 0.01). EphA2 STZ (n = 4) had a 26.4% decrease in epicardial capillary density compared with EphA2 (n = 6) control hearts after MI (p = 0.25). After infarction, EphA2 STZ (n = 4) hearts had 83.4% less epicardial capillaries than WT STZ (n = 5) hearts (p < 0.001). These results are depicted in Figure 7B.

### Echocardiography

#### STZ

Echocardiographic parameters for each of the 8 groups are presented in Table 1. Although there was no difference in EF between WT and EphA2-R-M mice, FS was depressed in EphA2-R-M mice and this difference was also present in hyperglycemic mice. Hyperglycemic EphA2-R-M mice also had reduced HR while WT mice had increased SBP and DBP. There were no differences in LV mass between uninjured normoglycemic and hyperglycemic EphA2-R-M and WT mice.

**Table 1:** **Cardiac function and blood pressure parameters**

***Parameter***	***EF***	***FS***	***LVID***_***d***_	***LVID***_***s***_	***LV vol***_***d***_	***LV vol***_***s***_	***HR***	***SBP***	***DBP***	***LV mass***
***WT (n=6)***	91 ± 3	60 ± 4	2.7 ± 0.3	1.1 ± 0.1	27.8 ± 7.2	2.5 ± 0.5	619 ± 98	132 ± 4	81 ± 3	102 ± 22
***EphA2-R-M (n=9)***	82 ± 4	49 ± 4^*^	2.7 ± 0.2	1.4 ± 0.2	27 ± 5.7	4.8 ± 1.5	636 ± 71	129 ± 4	77 ± 2	71 ± 15
***WT STZ (n=8)***	88 ± 2	57 ± 3	2.8 ± 0.2	1.2 ± 0.1	30.1 ± 4.7	3.6 ± 0.9	578 ± 98	185 ± 6*	107 ± 11*	69 ± 11
***EphA2-R-M STZ (n=18)***	78 ± 6	46 ± 6^●^	2.7 ± 0.2	1.5 ± 0.2	27.8 ± 5.6	6.1 ± 2.4	492 ± 142^†^	148 ± 4	91 ± 3	60 ± 15
***WT 4d (n=7)***	77 ± 1	45 ± 5	3.4 ± 0.4*	1.9 ± 0.3	51.0 ± 14.1*	13.5 ± 5.4*	658 ± 91	129 ± 8	73 ± 5	169 ± 51
***EphA2-R-M 4d (n-8)***	79 ± 3	46 ± 3	3.2 ± 0.1	1.7 ± 0.1	39.5 ± 2.2	8.5 ± 1.5	632 ± 93	126 ± 4	76 ± 3	94 ± 24^‡^
***WT STZ + 4d (n=7)***	55 ± 13^‡^	28 ± 8^‡^	3.3 ± 0.3	2.4 ± 0.5^‡^	44.9 ± 10.0	21.0 ± 9.1^‡^	602 ± 98	127 ± 6	73 ± 7	107 ± 32^‡^
***EphA2-R-M STZ + 4d (n=11)***	62 ± 12^¥^	33 ± 8^¥^	3.1 ± 0.7	2.1 ± 0.5	39.7 ± 18.6	15.4 ± 9.2	536 ± 80	130 ± 5	69 ± 2	81 ± 40

Echocardiography and blood pressure measures from conscious WT, EphA2-R-M, WT STZ, EphA2-R-M STZ, WT 4d, EphA2 3d, WT STZ + 4d, and EphA2-R-M STZ + 4d mice (p<0.05: *different from WT, ^†^different from EphA2-R-M, ^●^different from WT STZ, ^‡^ different from WT 4d, ^¥^different from EphA2-R-M 4d).

#### STZ + MI

EF and FS in both WT and EphA2-R-M mice 4 days post-MI were not different from their respective uninjured controls but diastolic diameter and volume as well as systolic volume are elevated in WT mice 4 days post-MI compared with uninjured control. Hyperglycemic WT and EphA2-R-M mice had depressed cardiac function 4 days post-MI compared with respective normoglycemic infarcted mice. Systolic diameter and volume were only elevated in WT mice There was no difference in LV mass in infarcted STZ mice but the EphA2-R-M mice did not exhibit proportionate compensatory hypertrophy demonstrated by the WT mice 4 days post-MI.

### qRT-PCR

#### STZ

**Figure 8:**
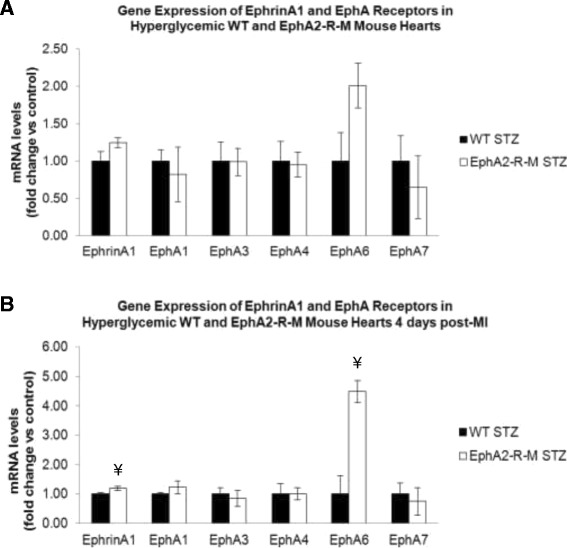
**Gene Expression of EphrinA1 and Eph Receptors in WT and EphA2-R-M STZ hearts before (A) and after (B) MI.** There were no significant differences between any of the genes tested in uninjured control hearts but ephrinA1and EphA6-R were elevated in EphA2-R-M hearts post-MI. ¥ Different from WT (p < 0.05).

EphrinA1 and EphA-R receptor gene expression was quantified using qRT-PCR mRNA levels for WT STZ mice (n = 4) and EphA2-R-M STZ mice (n = 5) (Figure 8). EphrinA1 ligand expression exhibited a non-significant trend to increase (1.24-fold; p = 0.08) in EphA2-R-M STZ hearts compared with WT STZ hearts. EphA6 also exhibited a non-significant trend to increase (2.01-fold, p = 0.06) in EphA2-R-M STZ hearts compared with WT STZ hearts.

#### STZ + MI

EphrinA1 and Eph Receptor gene expression was also quantified for WT STZ (5) and EphA2-R-M STZ (4) after infarction (Figure 8). Similar to uninjured STZ hearts, ephrinA1 and EphA6 were higher among EphA2-R-M STZ hearts after infarction. EphrinA1 was increased 1.19-fold (p < 0.05) and EphA6 was increased nearly 4.5-fold (p < 0.05) in EphA2-R-M STZ hearts after infarction.

### Western blotting

#### STZ

**Figure 9:**
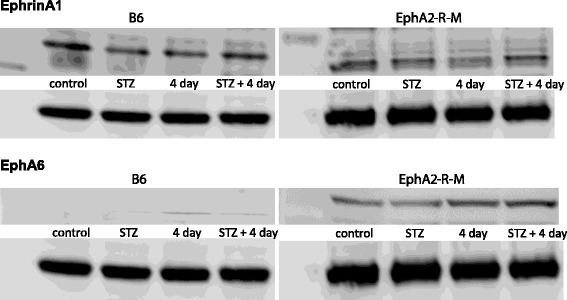
**Western blots.** Representative samples of left ventricles of uninjured and infarcted hearts from normoglycemic and hyperglycemic WT (left) and EphA2-R-M (right) mice probed with ephrinA1 (top) and EphA6-R (bottom) and normalized to GAPDH.

Representative blots for ephrinA1 and EphA6-R in WT mice (left) and EphA2-R-M mice (right) are shown in Figure 9. There were no statistically significant differences in expression of NF-κB, MMP-9, or p-mTOR/mTOR in response to STZ in either group (data not shown).

#### STZ + MI

Although not significant, there was a trend for reduced ephrinA1 (13%) expression in normoglycemic WT hearts post-MI and increased EphA6-R expression in both normoglycemic and hyperglycemic WT and EphA2-R-M hearts (11%) post-MI. There were no significant differences in NF-κB, MMP-9, or p-mTOR/mTOR expression between the two groups (data not shown).

## Discussion

Cardiomyocyte death post-MI starts immediately after coronary occlusion and occurs downstream from the occlusion. The therapeutic goal to decrease infarct size to less than 20% of the left ventricle has shown to improve long-term prognosis [23]. Early diagnosis and treatment are critical but presently inadequate. Administering a recombinant protein alone or in conjunction with grafting cells has reduced early injury as well as adverse remodeling and subsequent cardiac dysfunction [24–29]. Clinically, only reperfusion therapy with angioplasty or thrombolysis has been proven to reduce early injury. Although late remodeling cannot be significantly altered, it is managed with pharmacologic agents which sustain ventricular function but this merely delays the progression to heart failure. Current therapeutics, such as early myocardial revascularization after myocardial infarction, are geared toward minimizing cardiac myocyte necrosis and may modulate apoptosis and autophagy [30]. Research that explores the number and type of cells for grafting alone or in combination with gene and protein-based therapeutic strategies are aimed at augmenting the cardiac regenerative potential and promoting tissue salvage. Due to poor reproducibility and efficacy, as well as untoward side effects, none of these experimental paradigms have been successfully translated into clinical therapies. Due to these factors and the narrow window of opportunity between the initial cardiac insult and the cellular demise, developing newer therapies for “cellular resuscitation” are of paramount importance [31–33].

Diabetes has long been associated with greater incidence of myocardial infarction, inadequate healing, and susceptibility to further events. Hyperglycemia, the principal manifestation of all types of diabetes, results in perturbations in cellular metabolism, oxidative stress, exacerbated inflammation, and AGE-modified proteins that culminate in tissue damage, cardiac dysfunction, and impaired wound healing responses [34–42]. Sweeney et al. (2012) observed decreased glucose uptake and increased autophagy after acute I/R injury in STZ hearts, however, long term studies reveal detrimental effects on remodeling that ultimately compromise performance [43].

The EphA2-R has historically been known to regulate apoptosis in developing vasculature and nervous system and cell death during tumorigenesis [5,44–50], the EphA2-R also modulates vascular permeability, inflammation, and ischemic injury [11,12,14,51–59]. Knockdown of EphA2-R in HUVECs suppresses thrombin-induced phosphorylation of NF-κB, resulting in decreased ICAM-1 expression [59]. We observed increased sICAM-1 in serum of WT mice post-MI but this is blunted in hyperglycemic mice. Interestingly, sICAM-1 is decreased in hyperglycemic EphA2-R-M mice compared to normoglycemic mice and is not detected post-MI but is increased in hyperglycemic EphA2-R-M post-MI. EphrinA1 and EphA2 are both upregulated in response to inflammation [60–62]. Although we did not detect differences in NF-kB expression, we did observe decreased ephrinA1 expression in EphA2-R-M mice post-MI, and propose that the deficiency of EphA2-R prevents timely induction of inflammatory cascades necessary for mediating repair. Further, cardiac ephrinA1 expression exhibited an non-significant trend to increase slightly in hyperglycemic WT mice post-MI but not to the extent that they are expressed in uninjured normoglycemic mice, and is thus likely ineffective in eliciting the protective effects known to occur from exogenous administration of ephrinA1-Fc [13–15]. TNF-α, IL-1α, MIG, and TREM were only detectable in serum of hyperglycemic EphA2-R-M mice, and significant elevation of MCP-1, TIMP-1, and C5a, which typically correlate with the severity of injury, suggests exacerbated wound healing in EphA2-R-M mice [63–67]. Additionally, KC and M-CSF are reduced in hyperglycemic EphA2-R-M mice post-MI, implying poor monocyte adhesion and differentiation [63,68–70]. This is supported by the reduced leukocyte infiltration observed in this group, and although cardiac function is not worsened, the increased mortality in this group indicates that inadequate healing mechanisms acutely result in lethality. Indeed, plasma sICAM-1 level is associated with vascular inflammation and has been shown to correlate with increased risk of cardiovascular events [71,72], providing further support for the continuity of our findings.

We have previously shown that intramyocardial administration of ephrinA1-Fc reduces early injury in non-reperfused myocardium via reduced inflammatory cell infiltration and changes in gene expression of several EphA-Rs. The mechanisms by which this protection occurs can be multifaceted depending on the EphA receptors activated/inhibited and the expression profile of the various cells involved. Goichberg et al. (2012) showed ephrinA1-Fc enhanced motility of EphA2-expressing hCSCs, promoting infarct repair [14]. In adult mouse immortalized HL-1 cardiomyocytes, increased EphA2 expression and reduced phosphorylation following lithocholic acid treatment prevented doxazosin-induced apoptosis via inhibition of SHP-2 [73]. Clearly, the EphA2 receptor plays a vital role in myocardial tissue viability and more studies to understand the signaling pathways involved are warranted.

Expression of the EphA6 receptor gene decreases in response to injury and stays decreased with ephrinA1-Fc [13]. Previous work has shown it to be expressed in genital tubercle vascular endothelia and expression is regulated by HOXA13, a homeobox gene [74]. HOXA13 is also a transcriptional regulator of appropriate vessel morphology and function of the developing placenta [75]. In a recent study, we showed that the EphA6-R localizes to the vasculature and increased in response to injury in EphA2-R-M mouse hearts [76], suggesting that EphA6-R compensates for the absence of EphA2-R. In the current study, only EphA6 was different between uninjured normoglycemic and hyperglycemic WT and EphA2-R-M mouse hearts. While not altered significantly by hyperglycemia at 4 days post-MI in either strain, it was elevated post-MI in all groups. EphA6 is expressed in adult human monocytes and coronary artery endothelial cells [77]. Therefore, given that several ephrins and Eph receptors are upregulated during injury in various cell types, they may play a role in chemotaxis and extravasation and provide insights into the mechanisms of inflammatory diseases and damage resolution [78–80].

## Conclusions

This research ratifies our understanding of the negative impact of hyperglycemia on the form and function of the wild type heart. Hyperglycemia leads to decreased survival following MI, impaired compensatory hypertrophy of cardiomyocytes, and increased interstitial fibrosis of the left ventricle. Superimposing hyperglycemia on the infarcted transgenic EphA2-R-M mouse impaired the response to injury and significantly increased mortality. These studies provide the baseline data needed to begin unraveling the role of EphA2-R in modulating inflammation and evaluate the potential impact of ephrinA1-Fc use in the treatment of MI in the hyperglycemic myocardium.
